# Supplementary material for: Progression and Dissemination of Pulmonary Mycobacterium Avium Infection in a Susceptible Immunocompetent Mouse Model
Source: Int J Mol Sci. 2022 May 26;23(11):5999. doi: 10.3390/ijms23115999 (PMC9181083; doi:10.3390/ijms23115999)
Supplement: Supplementary file 1 [file ijms-23-05999-s001.zip › Supplemental Figure and legends-merged IJMS.pdf]

Supplemental figures and legends

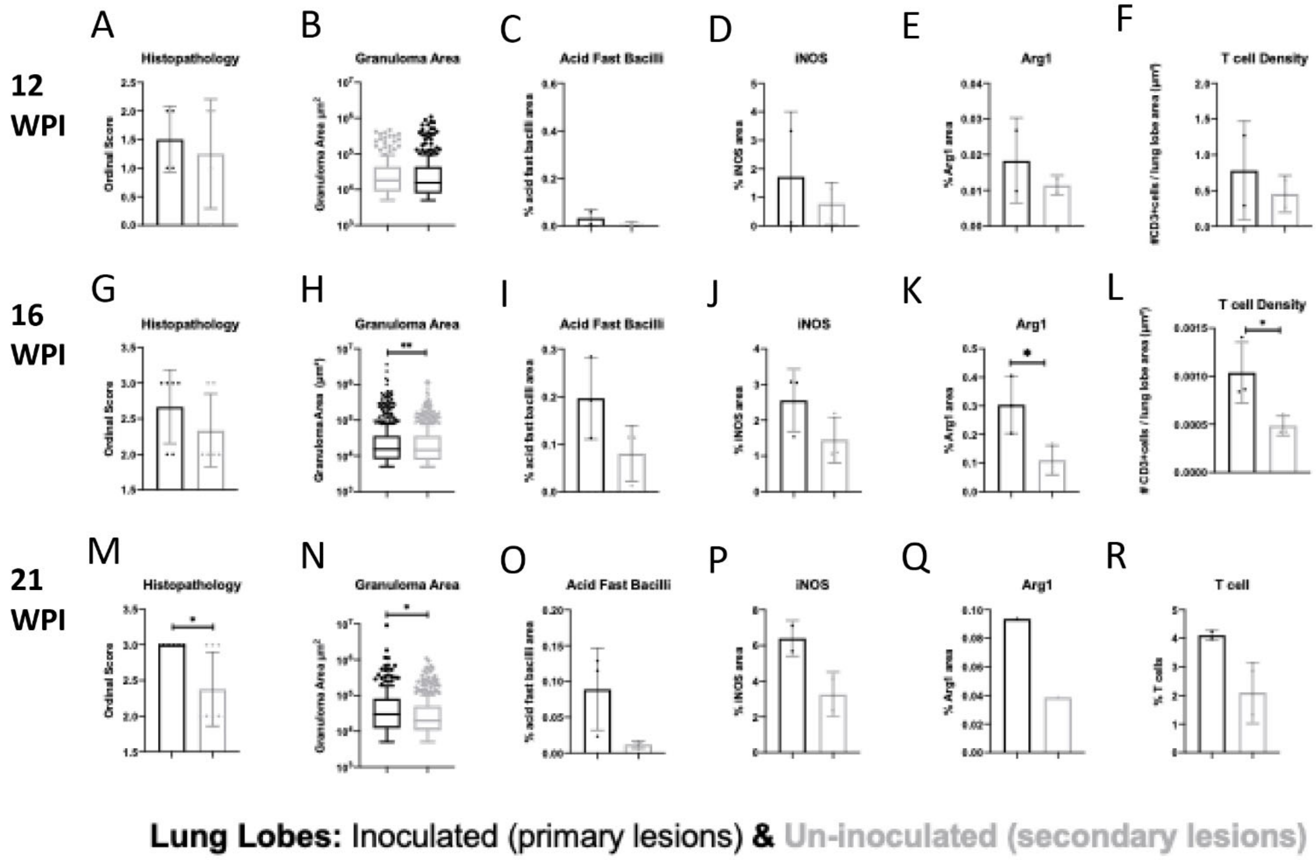

Supplemental Figure S1. Comparison between primary lesions and secondary lesions following unilateral left mainstream intrabronchial inoculation at 12- (A-F), 16- (G-L) and 21- (M-R) weeks post-infection (wpi). **A:** Histopathologic scores. **B:** Lesion area. **C:** M. *av* Acid fast bacilli (AFB) area quantification (AQ). **D:** iNOS AQ. **E:** Arg1 AQ. **F:** T cell density. **G:** Histopathologic scores. **H:** Lesion area. **I:** AFB AQ. **J:** iNOS AQ. **K:** Arg1 AQ. **L:** T cell density. **M:** Histopathologic scores. **N:** Lesion area. **O:** AFB AQ. **P:** iNOS AQ. **Q:** Arg1 AQ. **R:** CD3+ AQ. For A (N=4), G (N=6), and M (N=8), each data point represents a single tissue section. For B (N=257 [inoculated]; N=231 [uninoculated]), H (N=662 [inoculated]; N=913 [uninoculated]), and N (N=179 [inoculated]; N=306 [uninoculated]), each data point represents a single lesion. For C-F (N=2), I-L (N=3), O-R (N=2), each data point represents a single animal. Data are expressed as means ± SD or with Tukey whiskers. Asterisks
denote P values with Mann Whitney test (*) or Student's unpaired T test (*-*).
$*P < 0.05$, $**P < 0.005$, $***P < 0.0005$, and $****P$

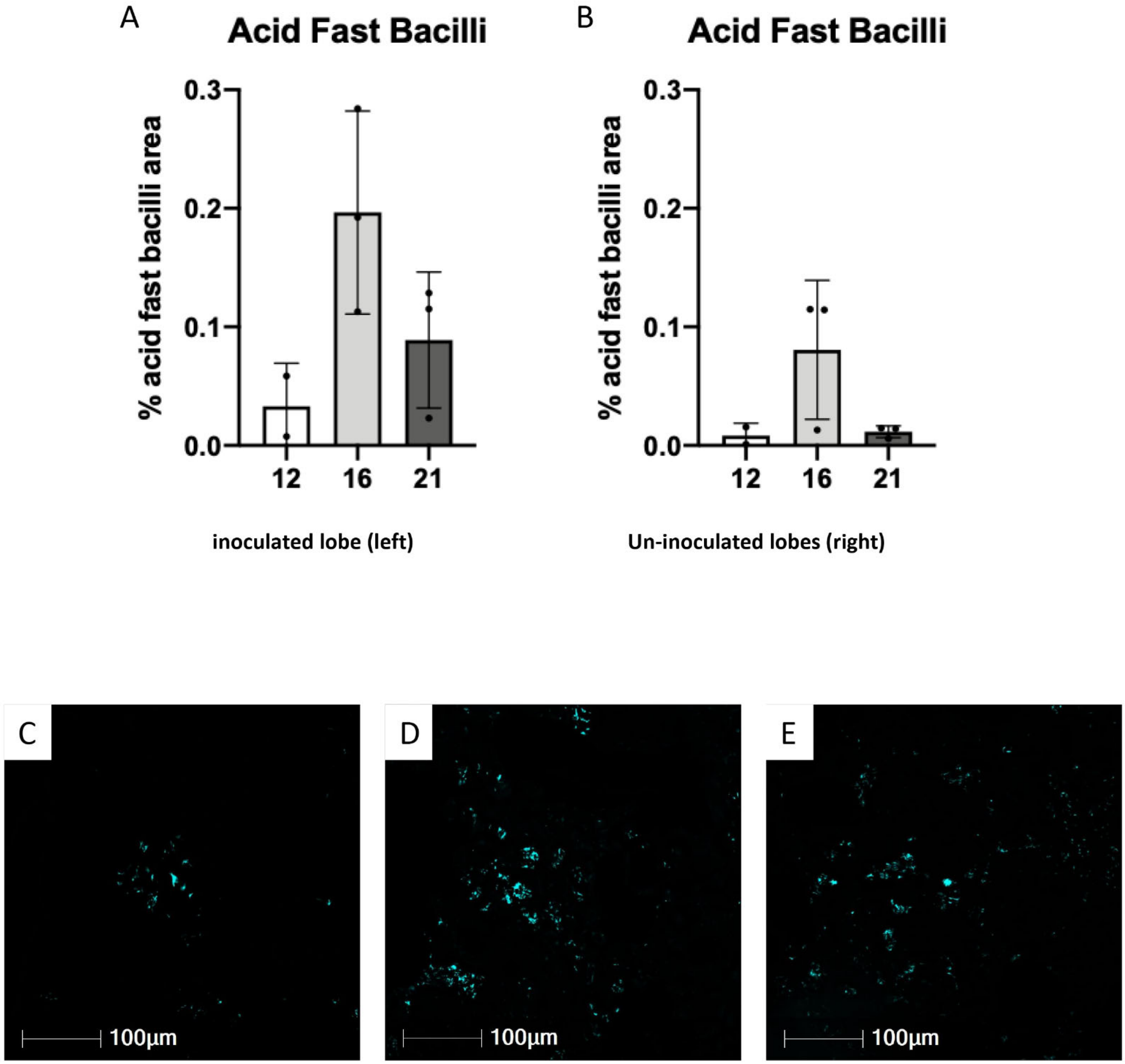

Supplemental Figure S2. Mycobacterial quantification and visualization via Auramine O-Rhodamine B Acid Fast Bacilli (AFB) staining following unilateral left mainstream intrabronchial inoculation at 12-, 16-, and 21-weeks post infection (wpi). **A-B**: AFB Area Quantification (AQ), at 12, 16, and 21 wpi; **A.** Inoculated, **B.** Un-inoculated. **C-E:** Representative raw fluorescent AFB stained lung sections from the un-inoculated lung lobes; **C.** 12 wpi, **D.** 16 wpi, **E.** 21 wpi. C-E visualized with Halo Image Analysis software v3.2 (Indica Labs, Corrales, NM) with analysis performed on AFB stained whole slide images. For A-B, N=2 (12 wpi), N=3 (16 wpi), N=3 (21 wpi). Original magnification, 200x. Scale bars: 100 µm (C-E).

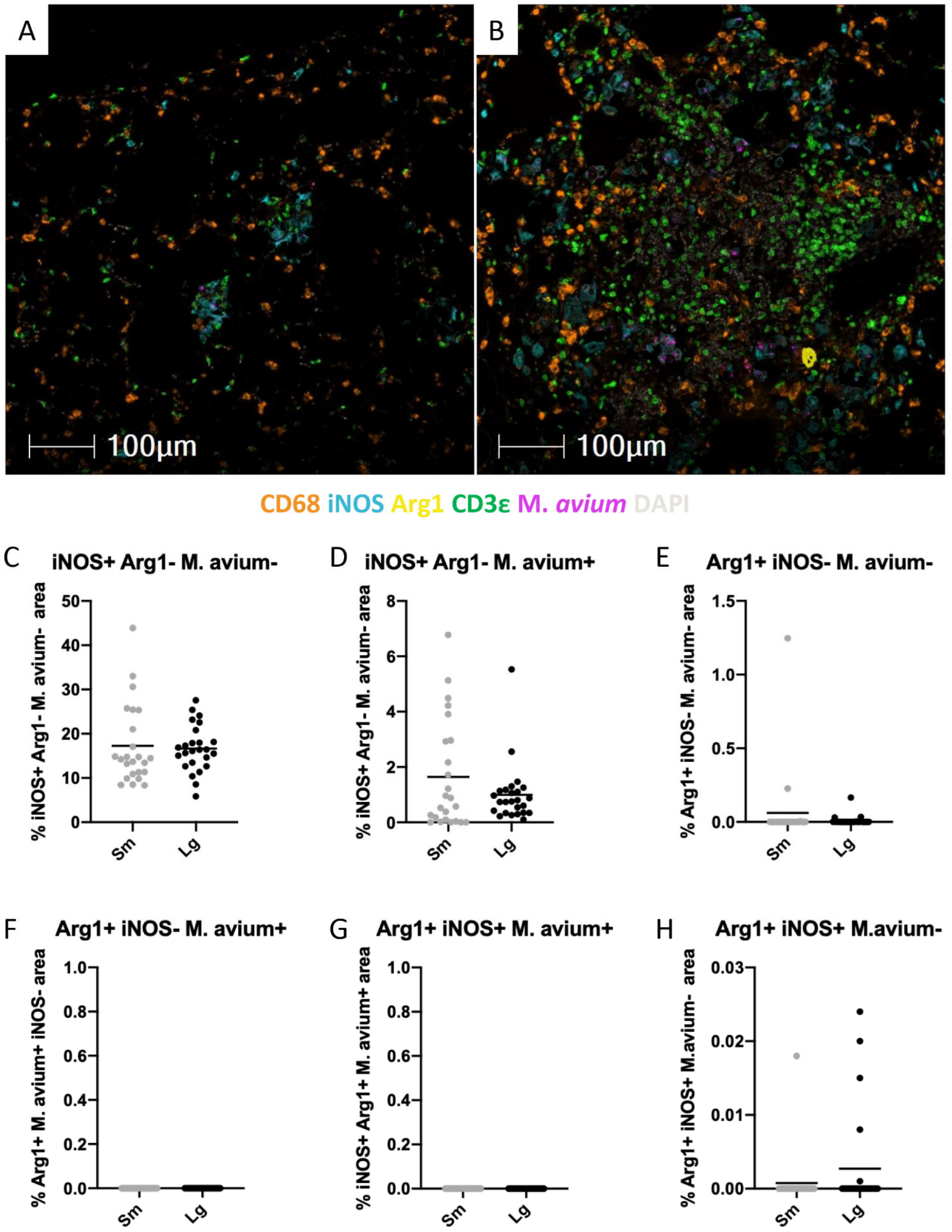

Supplemental Figure S3. Characterization of macrophage populations in secondary lesions at 12 weeks-post infection (wpi) following unilateral left mainstream intrabronchial M. *avium* inoculation in B6.Sst1S mice. **A:** Representative raw fluorescent multiplex immunohistochemical (fmIHC) image of small secondary lesion. B: Representative raw fluorescent mIHC image of large secondary lesion. A-B visualized with Halo Image

Analysis software v3.2 (Indica Labs, Corrales, NM) with analysis performed on fluorescent labeled whole slide images. C-H Area quantification (AQ) of iNOS, Arg1 and M. *avium* overlay in small and large granulomatous lesions; C**.** iNOS+/Arg1-/M. *avium*- AQ. D**.** iNOS+/Arg1-/M. *avium*+ AQ, E**.** Arg1+/iNOS-/M. *avium*- AQ, F**.** Arg1+/iNOS-/M. *avium*+ AQ, G**.** Arg1+/iNOS+/M. *avium*+ AQ, H**.** Arg1+/iNOS+/M. *avium*- AQ. For C-H, each data point represents a single lesion and N=24 (small lesions), N=25 (large lesions). Data are expressed on scatter dot plots with medians. Original magnification 100x (**A**). Scale bars: 100 µm (A-B).

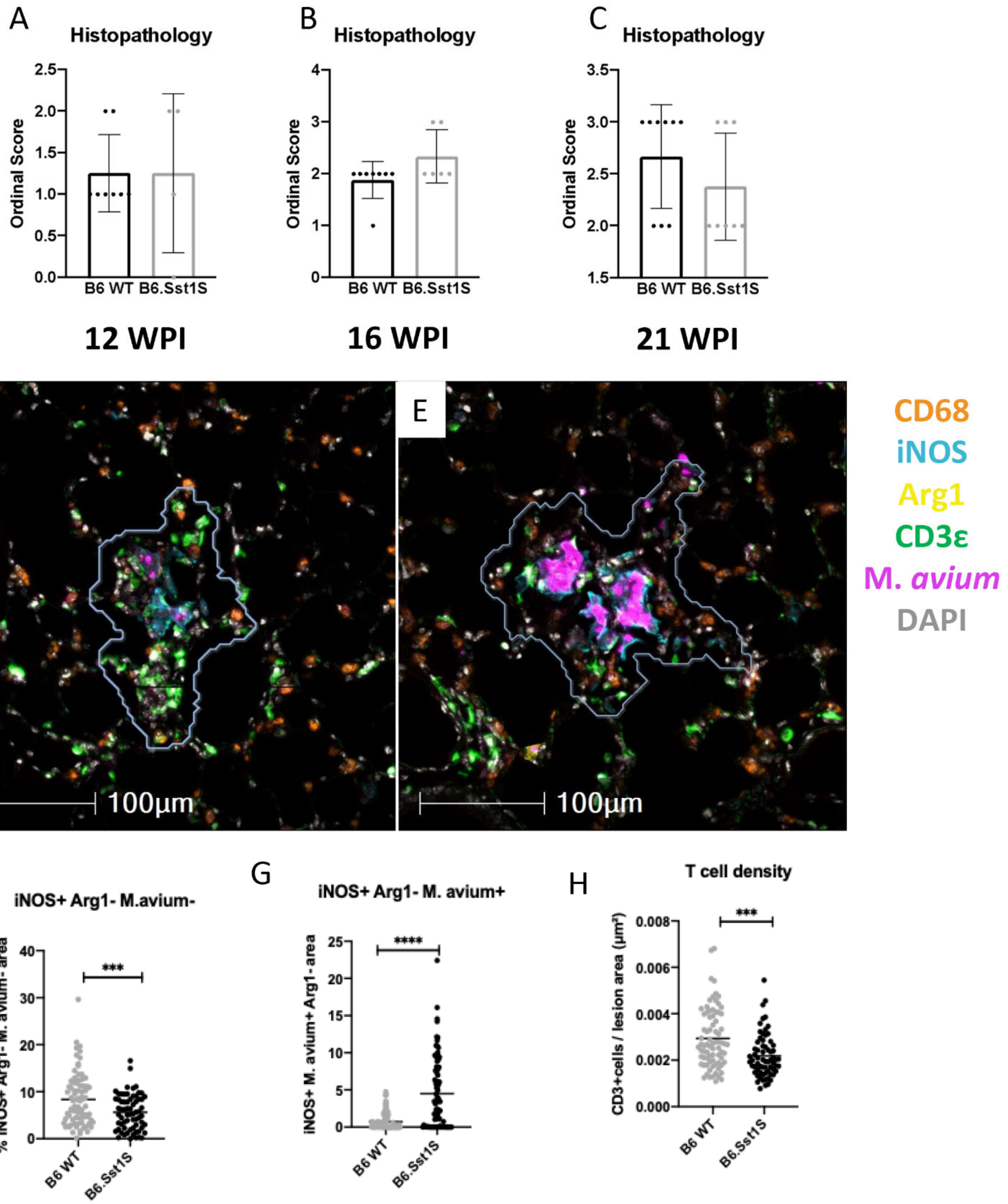

Supplemental Figure S4. B6 WT vs. B6.Sst1S ordinal histopathology comparisons following unilateral left mainstream intrabronchial inoculation at 12-, 16-, and 21-weeks post-infection (wpi) and the impacts of *sst1*-S

in small secondary lesions at 16 wpi. **A:** Histopathology scores at 12 wpi. **B:** Histopathology scores at 16 wpi. **C:** Histopathology scores at 21 wpi. **D:** Representative raw fluorescent multiplex immunohistochemical (fmIHC) image of B6 WT small secondary lesion. **E:** Representative raw fmIHC image of B6.Sst1S small secondary lesion. D-E visualized with Halo Image Analysis software v3.2 (Indica Labs, Corrales, NM) with analysis performed on fluorescent labeled whole slide images. **F-G**: iNOS, Arg1, M. avium Area quantification (AQ) immunoreactivity in small B6 WT and B6.Sst1S lesions; **F.** iNOS+ Arg1- M. avium- AQ, **G.** iNOS+ Arg1- M. avium+ AQ, **H.** T cell lesion density in B6 WT and B6.Sst1S small secondary lesions. For A-C, each data point represents one tissue section; N=8 (12 & 16 wpi B6 WT), N=4 (12 wpi B6.Sst1S), N=6 (16 wpi B6.Sst1S), N=9 (21 wpi B6 WT), N=8 (B6.Sst1S). For F-G, percentage area immunoreactivity was calculated in relation to area of given lesion. Each data point represents one lesion; N=78 (B6 WT), N=67 (B6.Sst1S). Data are expressed as means ± SD or with Tukey whiskers. Asterisks denote P values with Mann Whitney test (*) or Student's unpaired T test (*-*). $*P < 0.05$, $**P < 0.005$, $***P < 0.0005$, and $****P$. Original magnification, 100x (**D** and **E**). Scale bars: 100 μm (E-D).

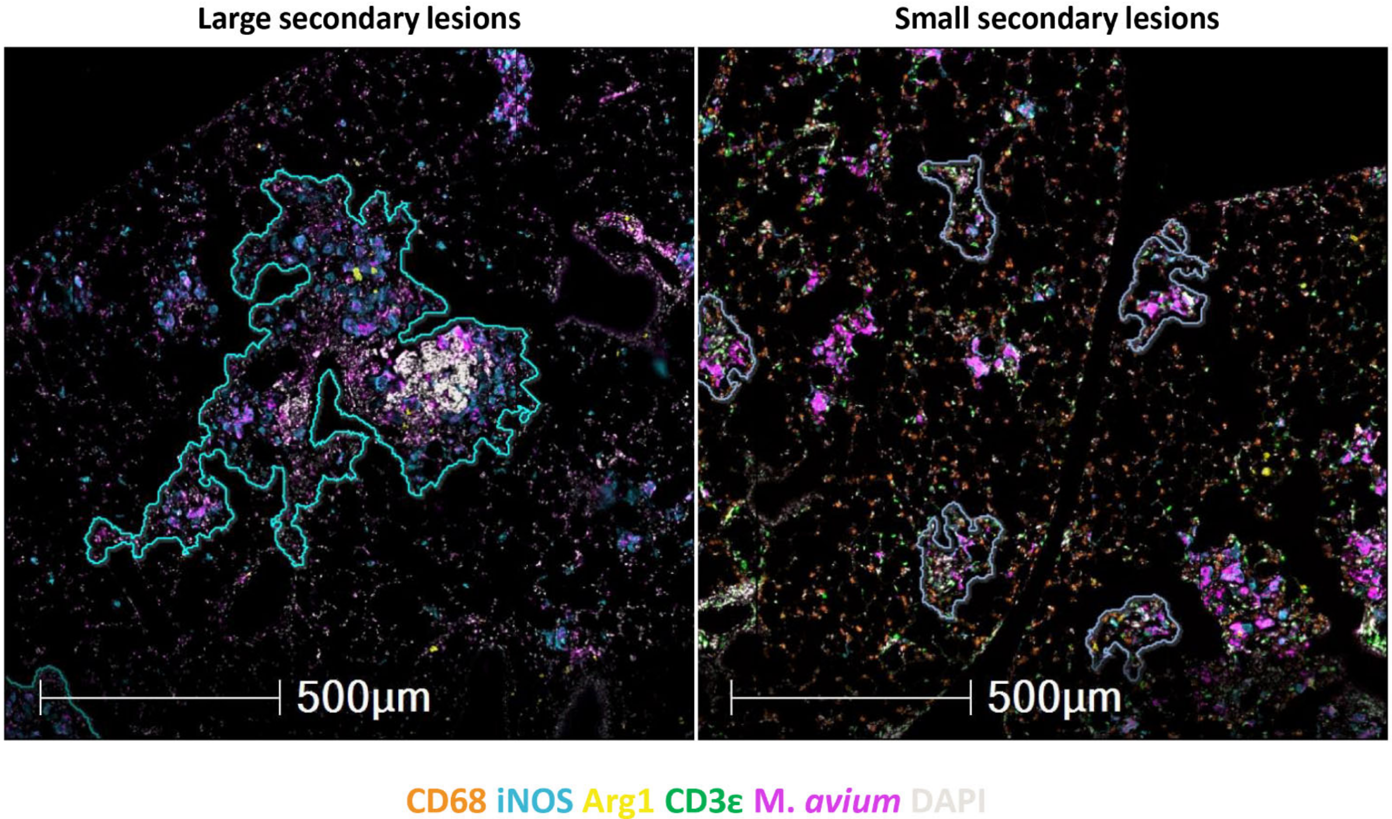

Supplemental Figure S5. Representative large (left panel) and small (right panel) secondary lesions as identified by a random forest classifier that detected iNOS, Arg1, M. *avium* (M. *av*), and interspersed CD3e+ cells in the uninoculated lung lobes of B6.Sst1S mice at 16 wpi following intrabronchial inoculation with Mycobacterium *avium* (M. *av).* Large secondary lesions represented the top 10 percentile of lesions by area for each given specimen. Small secondary lesions represented lesions between 50-60th percentile lesions by area for each given specimen. Lesions were excluded if they were directly affiliated with a bronchiole. Images visualized with Halo Image Analysis software v3.2 (Indica Labs, Corrales, NM) with analysis performed on fluorescent labeled whole slide images. Original magnification 50x. Scale bars: 500 μm.
