# Supplementary material for: Progression and Dissemination of Pulmonary Mycobacterium Avium Infection in a Susceptible Immunocompetent Mouse Model
Source: Int J Mol Sci. 2022 May 26;23(11):5999. doi: 10.3390/ijms23115999 (PMC9181083; doi:10.3390/ijms23115999)
Supplement: Supplementary file 1 [file ijms-23-05999-s001.zip › Supplemental Tables S1-S6 merged.pdf]

Supplemental Table S1. Pulmonary Histopathology Ordinal Score Summary (intranasal inoculation)					
Weeks post-infection (wpi) and inoculation dosage	Animal ID	Slide #	Ordinal Score	strain	sex
4 wpi 10^6	C1-1	1	0	B6.Sst1S	F
		4	1		
		7	1		
	C1-2	1	1	B6.Sst1S	F
		4	1		
		7	1		
	C1-3	1	0	B6.Sst1S	M
		4	1		
		7	1		
	C5-1	1	0	B6.Sst1S	M
		4	0		
		7	0		
	C5-2	1	1	B6.Sst1S	M
		4	1		
		7	1		
	C5-3	1	1	B6.Sst1S	M
		4	2		
		7	2		
8 wpi 10^6	4-1	1	2	B6.Sst1S	M
		4	2		
		7	2		
	4-2	1	1	B6.Sst1S	M
		4	2		
		7	2		
10 wpi 10^6	2-1	1	2	B6.Sst1S	F
		4	2		
		7	2		
	2-2	1	1	B6.Sst1S	F
		4	2		
		7	2		
	3-1	1	2	B6.Sst1S	F
		4	2		

		7	2		
12 wpi 10^6	3-2	1	2	B6.Sst1S	F
		4	2		
		7	2		
	3-3	1	2	B6.Sst1S	F
		4	2		
		7	2		
	3-4	1	2	B6.Sst1S	F
		4	2		
		7	2		
	14-2	1	2	B6.Sst1S	F
		4	3		
		7	2		
	14-3	1	2	B6.Sst1S	F
		4	2		
		7	2		

**Supplemental Table S2**. **Ordinal Grading Score: *Mycobacterium avium* intranasal and intrabronchiolar inoculation**	
0	No significant findings observed.
1	Multifocal lymphohistiocytic perivascular and peribronchiolar inflammation; minimal granulomatous pneumonia(<10% parenchyma).
2	Multifocal lymphohistiocytic perivascular and peribronchiolar inflammation; mild-to-moderate granulomatous pneumonia with rare intralesional single cell necrosis and neutrophils (>10-30% parenchyma).
3	Multifocal lymphohistiocytic perivascular and peribronchiolar inflammation; moderate-to-marked granulomatous pneumonia with mild intralesional single cell necrosis and neutrophilic infiltrate, sporadic multinucleated macrophages) and mild intralesional pulmonary fibrosis (>30-50% parenchyma)

**Supplemental Table S3. Pulmonary Histopathology Ordinal Score Summary (Left mainstream intrabronchial inoculation)**

Weeks Post-Infection (wpi) and inoculation dosage	Animal ID	Depth of Block (mm from ventral surface)	Strain	Left lung (inoculation site)	Right lungs (un-inoculated)
12 wpi 10^5	1	1	WT	2	2
		2		2	2
	2	1		1	1
		2		1	1
	3	1		1	1
		2		1	1
	4	1		2	1
		2		2	1
	13	1	B6.Sst1 S	2	2
		2		2	2
	14	1		1	0
		2		1	1
	15	1		0	0
		2		0	0
16 wpi 10^5	5	1	WT	3	2
		2		3	2
	6	1		3	2
		2		3	2
	7	1		2	2
		2		2	1
	8	1		2	2
		2		2	2
	16	1	B6.Sst1 S	3	3
		2		3	3
	17	1		3	2
		2		3	2
	18	1		0	0

		2		0	0
	19	1		2	2
		2		2	2
21 wpi 10^5	9	1	WT	2	2
		2		2	2
		3		2	2
	10	1		3	3
		2		3	3
		3		3	3
	11	1		2	3
		2		3	3
		3		3	3
	12	1		0	0
		2		0	0
		3		0	0
	20	1	B6.Sst1S	3	3
		2		3	3
		3		3	2
	21	1		3	2
		2		3	2
		3		3	2
	22	1		3	2
		2		3	3

Supplemental Table S4. Optimized conditions for manual fmIHC (5-plex)

**Order**	AR Conditions	Rabbit derived primary antibody (Ab)	Manufacture Catalog #	Primary Ab titration	Primary Ab incubation length	TSA-conjugated Fluorophore	Manufacture Catalog #	Opal Titration

1	90 degrees for 15min	Arginase-1 (Arg1)	Cell Signaling D4E3M	1:100	1 hour at room temperature	570	Akoya Biosciences, FP1488001KT	1:100
2	90 degrees C for 15min	Inducible nitric oxide synthase (iNOS)	abcam ab15323	1:200	Overnight at 4 degrees C	480	Akoya Biosciences, FP1500001KT	1:200
3	95 degrees C for 40 minutes & AR9 buffer	CD68	Fisher Scientific PA5-32330	1:100	Overnight at 4 degrees C	620	Akoya Biosciences, FP1495001KT	1:100
4	95 degrees C for 40 minutes	CD3e	ThermoFisher MA5-14524	1:100	3 hours at room temperature	520	Akoya Biosciences, FP1487001KT	1:200
5	90 degrees for 15min	Mycobacterium *tuberculosis*	Biocare Medical CP140A	1:200	1 hour at room temperature	780	Akoya Biosciences, FP1501001KT	1:12.5

Supplemental Table S5. Optimized conditions for automated fmIHC (6-plex)

**Order**	Pre-Treatment-duration	Rabbit derived primary antibody (Ab)	Manufacture, Catalog #	Primary Ab titration	Primary Ab incubation length and temper-ature	TSA-conjugated Fluoro-phore	Manu-facture Catalog #	Opal Titra-tion

1	CC1-95-64 minutes	Arginase-1 (Arg1)	Cell Signaling Technology D4E3M	1:100	24 min 37° C	570	Akoya Biosciences, FP1488001KT	1:100
2	CC1-95-64 minutes	Inducible nitric oxide synthase (iNOS)	Abcam ab15323	1:200	32 min 37° C	480	Akoya Biosciences, FP1500001KT	1:100
3	CC1-95-32 minutes	CD68	Abcam ab12512	1:100	60 min 37° C	690	Akoya Biosciences, FP1497001KT	1:100
4	CC2-95-64 minutes	CD4	Cell Signaling Technology 25229	1:50	60 min 37° C	620	Akoya Biosciences, FP1495001KT	1:100
5	CC1-95-64 minutes	CD3e	Thermo Fisher MA5-14524	1:100	60 min 37° C	520	Akoya Biosciences, FP1487001KT	1:100
6	CC1-95-64 minutes	Myco-bacterium *tuberculosis*	Biocare Medical CP140A	1:100	60 min 37° C	780	Akoya Biosciences, FP1501001KT	1:25

Supplemental Table S6. Optimized conditions for automated YFP fmIHC (5-plex)

**Order**	Pre-Treat-ment-duration	Rabbit derived primary antibody (Ab)	Manufac-ture Catalog #	Primary Ab titration	Primary Ab incubation length	TSA-conjugated Fluoro-phore	Manufacture Catalog #	Opal Titration

1	CC1-95- 64 minutes	Arginase-1 (Arg1)	Cell Signaling Technology D4E3M	1:100	24 min 37° C	520	Akoya Biosciences, FP1487001KT	1:200
2	CC1-95- 64 minutes	Mycobacterium *tuberculosis*	Biocare Medical CP140A	1:100	60 min 37° C	570	Akoya Biosciences, FP1488001KT	1:125
3	CC1-95- 64 minutes	GFP	Invitrogen A11122	1:100	24 min 37° C	480	Akoya Biosciences, FP1500001KT	1:250
4	CC1-95- 64 minutes	Inducible nitric oxide synthase (iNOS)	Abcam ab15323	1:200	32 min 37° C	690	Akoya Biosciences, FP1497001KT	1:75
5	CC1-95- 64 minutes	CD3e	Thermo Fisher MA5-14524	1:100	60 min 37° C	620	Akoya Biosciences, FP1495001KT	1:100
